# Platelet‐Like Fusogenic Liposome‐Mediated Targeting Delivery of miR‐21 Improves Myocardial Remodeling by Reprogramming Macrophages Post Myocardial Ischemia‐Reperfusion Injury

**DOI:** 10.1002/advs.202100787

**Published:** 2021-06-17

**Authors:** Haipeng Tan, Ya'nan Song, Jing Chen, Ning Zhang, Qiaozi Wang, Qiyu Li, Jinfeng Gao, Hongbo Yang, Zheng Dong, Xueyi Weng, Zhengmin Wang, Dili Sun, Wusiman Yakufu, Zhiqing Pang, Zheyong Huang, Junbo Ge

**Affiliations:** ^1^ Department of Cardiology Zhongshan Hospital Fudan University Shanghai Institute of Cardiovascular Diseases 180 Fenglin Road, Xuhui District Shanghai 20032 P. R. China; ^2^ School of Pharmacy Fudan University Key Laboratory of Smart Drug Delivery Ministry of Education 826 Zhangheng Road, Pudong New Area Shanghai 201210 P. R. China

**Keywords:** Biomimetic, macrophages, membrane fusion, miRNAs, monocytes, myocardial ischemia‐reperfusion injury, platelets

## Abstract

Inflammatory modulations focusing on macrophage phenotype are promising candidates to promote better cardiac healing post myocardial ischemia‐reperfusion (MI/R) injury. However, the peak of monocyte/macrophage recruitment is later than the time when enhanced permeability and retention effect disappears, which greatly increases the difficulty of reprogramming macrophages through systemic administration. Meanwhile, the inability of nanomaterials to release their contents to specific intracellular locations through reasonable cellular internalization pathways is another obstacle to achieving macrophage reprogramming. Here, inspired by the increase in circulating platelet‐monocyte aggregates in patients′ post‐MI/R and the high efficiency of fusogenic liposomes to deliver contents to the cytoplasm of target cells, a platelet‐like fusogenic liposome (PLPs) is constructed. Under the coating of PLPs, mesoporous silica nanospheres with a payload of miR‐21, an anti‐inflammatory agent, can be specifically delivered to inflammatory monocytes in the blood circulation of MI/R induced mice. Then it directly enters the cytoplasm of monocytes through membrane fusion, thereby realizing the reparative reprogramming of the inflamed macrophages derived from it. In vivo administration of the resulting formula can effectively preserve the cardiac function of mice undergone MI/R. Minimal invasiveness and biological safety make this nano‐platform a promising approach of immunotherapy.

## Graphical abstract

A platelet‐like fusogenic liposome is successfully developed. It mimics the interaction between platelets and monocytes/macrophages as a targeting strategy, and can deliver miR‐21 directly to the cytoplasm of monocytes/macrophages through membrane fusion, and then achieve macrophage reprogramming. In vivo administration of the resulting formula can effectively preserve the cardiac function of mice undergone MI/R.

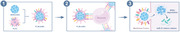

## 1 Introduction

Cardiovascular diseases, especially myocardial infarction (MI), and their related complications are leading causes of death worldwide.^[^ ^1^ ^]^ Timely reperfusion of patients with MI through percutaneous coronary intervention can significantly reduce their acute mortality, but a large proportion of the patients eventually progress to heart failure.^[^ ^2^ ^]^ A pivotal stage in the process of cardiac remodeling after myocardial ischemia‐reperfusion (MI/R) injury is the robust inflammatory cascade.^[^ ^3^ ^]^ The early inflammatory activation lays the foundation for the later reparative phase, but optimal healing post‐MI requires appropriate and timely resolution of the inflammation.^[^ ^4^ ^]^ Inflammatory macrophages (M1) enter the injured area at an early stage, secrete various cytokines and phagocyte debris.^[^ ^5^, ^6^ ^]^ Several studies have demonstrated that their timely switch to a reparative phenotype (M2) is crucial for the resolution of the inflammation.^[^ ^6^, ^7^, ^8^, ^9^ ^]^ Macrophages with distinct phenotypes are also involved in the angiogenesis process at different specific stages.^[^ ^10^, ^11^ ^]^ A recent study showed that M1 mainly promotes the sprouting of blood vessels, while M2 mainly promotes the maturation and quiescence of newly sprouting vessels.^[^ ^11^ ^]^ Long‐term exposure to M1 will cause the already formed blood vessels to regression, which indicates that promoting the timely reparative polarization of inflammatory macrophages after MI/R is also crucial for angiogenesis.^[^ ^11^ ^]^

In recent years, numerous studies, which are aimed at precisely regulating the polarization state of macrophages in the repair process post‐MI/R, have been carried out in full swing, and gene therapy is one of the most promising directions. As a powerful gene silencing tool, miRNA mainly regulates target mRNA by combining their incomplete complementarity of 3′‐unmodified regions in the cytoplasm, and RNA molecules have been widely studied as a powerful component of de novo synthesis of nanoparticles.^[^ ^12^, ^13^ ^]^ However, there are still three major obstacles to the success of miRNA therapy aimed at regulating the phenotype of macrophages. First, miRNAs and its mimics are easily degraded by RNase in body fluids and natural environment. Second, in previous studies aimed at regulating the polarization state of macrophages, the intervention time point was usually 2 days post‐MI/R or later, at which time the number of macrophages in the infarct begins to increase and the cells become influential.^[^ ^14^, ^15^ ^]^ However, the enhanced permeability and retention (EPR) effect has been significantly diminished at 24 h post‐MI/R.^[^ ^16^, ^17^ ^]^ The mismatch in time window makes most systematic delivery strategies, which rely on the EPR effect to passively target macrophages less successful.^[^ ^6^, ^16^, ^18^ ^]^ At last, nano‐formulations cannot deliver the miRNAs into the cytoplasm of macrophages in an effective way, so that miRNA cannot reach an effective therapeutic concentration in recipient cells.

Here, we developed an active and efficient miRNA targeted delivery platform, which aims to perform anti‐inflammatory reprogramming of inflammatory macrophages post‐MI/R. Considering the high efficiency of promoting reparative polarization of macrophages, miR‐21 was selected as the effector molecule of our nano‐formulation.^[^ ^19^, ^20^, ^21^ ^]^ First, in view of the biological protection and carrying efficiency of miRNAs, we used mesoporous silica nanospheres (MSNs) to load miRNAs by calcium silicate sealing.^[^ ^22^ ^]^ Its miRNA loading efficiency reaches ≈20%, and it can effectively protect miRNAs from degradation in vivo.^[^ ^22^ ^]^ Second, inspired by the increase of circulating monocyte‐platelet aggregates of patients with acute coronary syndrome, platelets (PLTs) bionic technology has become a potential navigation technology to endow the nano‐platform proactive targeting ability to monocytes/macrophages post‐MI/R.^[^ ^23^, ^24^, ^25^ ^]^ The binding between P‐selectin and P‐selectin glycoprotein ligand‐1 (PSGL‐1) is the main mediator of the interaction between PLTs and monocytes, and studies have shown that PSGL‐1 is highly expressed on circulating Ly6C+ monocytes, which is the main source of inflammatory macrophages in the area of MI.^[^ ^24^, ^26^, ^27^ ^]^ Finally, taking conventional lipid nanoparticles as carriers for RNAs delivery will leading to degradation of most administered RNAs by lysosomes through endocytosis and only ≈1–2% of that can be dispersed into the cytoplasm.^[^ ^28^, ^29^ ^]^ We adopted a previously reported method for preparing fusogenic liposomes that can impart membrane fusion properties to liposomes.^[^ ^30^ ^]^ This helps prevent liposomes from being endocytosed by recipient cells, but instead delivers its content directly to the cytoplasm through membrane fusion.

**Figure 1:**
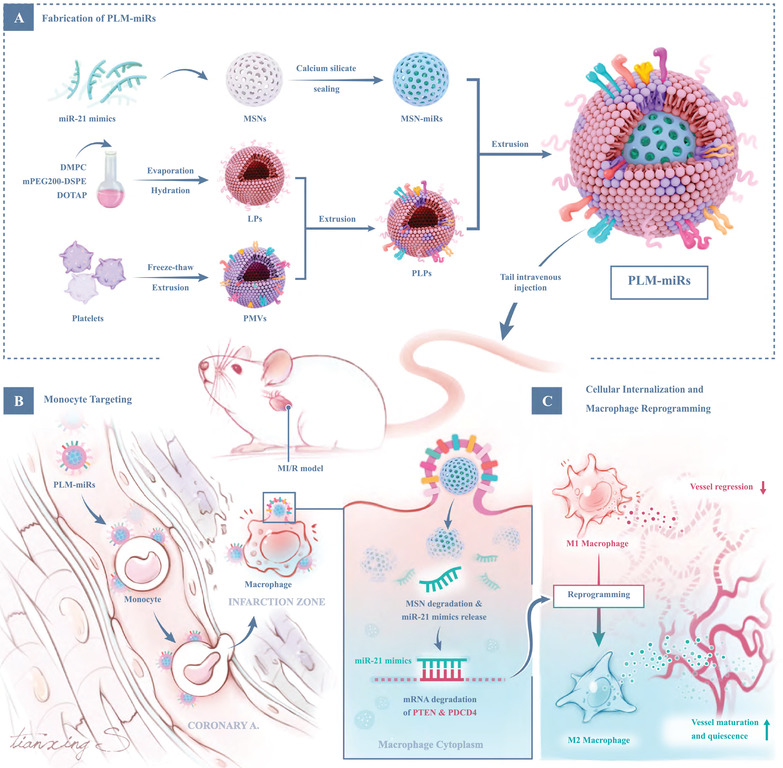
Schematic illustration of PLM‐miRs integrating active monocyte/macrophage targeting and high miR‐21 delivery efficiency. A) Fabrication of PLM‐miRs. MSNs are fully loaded with miR‐21 through calcium silicate sealing as the core of PLM‐miRs. The extruded LPs and PMVs are mixed and then extruded to synthesize PLPs as the shell of PLM‐miRs. B) Monocyte targeting. PLM‐miRs bind to monocytes that are chemotaxis to the injured heart in the blood circulation of MI/R induced mice, and are carried to the injured area. Monocytes evolve into macrophages after reaching local tissues. C) Cellular internalization and macrophage reprogramming effect of PLM‐miRs. PLM‐miRs deliver MSN‐miRs into the cytoplasm of monocytes/macrophages through membrane fusion. With the degradation of MSNs, the released miR‐21 mediates the mRNA degradation of target genes PTEN and PDCD4, leading to reparative reprogramming of inflammatory macrophages. The reprogramming effect reduces the regression of sprouting blood vessels caused by prolonged exposure to M1, and the obtained M2 promotes the maturation and quiescence of sprouting blood vessels.

Overall, we aim to design a more proactive targeting delivery platform to monocytes/macrophages post‐MI/R. This delivery system consists of MSNs loaded with miR‐21 as the core and a fusion membrane of platelet membrane and cationic liposome, namely platelet‐like fusogenic liposomes (PLPs), as the outer shell (**Figure** **1**A). After intravenous injection, our formula is first carried by the recruited Ly6C+ monocytes to the injured area through mimicking the aggregation of PLTs and monocytes (Figure 1B). Then, the miRNAs it carries are directly released into the cytoplasm of recipient monocytes through membrane fusion (Figure 1C). And then it reprograms these monocyte‐derived inflamed macrophages to a reparative phenotype in the acute inflammatory phase post‐MI/R (Figure 1C).

## 2 Results

### 2.1 Fabrication and Characterization of PLM‐miRs

**Figure 2:**
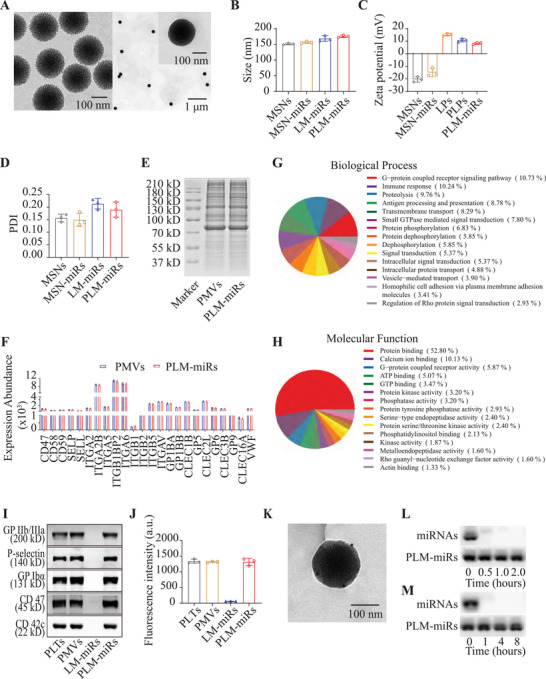
Preparation and characteristics of PLM‐miRs. A) TEM of MSNs (left) and PLM‐miRs (right). B) Size of MSNs, MSN‐miRs, LM‐miRs, and PLM‐miRs (*n* = 3). C) Zeta potential of MSNs, MSN‐miRs, LPs, PLPs, and PLM‐miRs (*n* = 3). D) Polymer dispersity index of MSNs, MSN‐miRs, LM‐miRs, and PLM‐miRs (*n* = 3). E) SDS‐PAGE analysis of PMVs and PLM‐miRs. F) Expression abundance of proteins associated with platelet adhesion and immune escape properties for PMVs and PLM‐miRs (*n* = 3). G) Classification of PLM‐miRs proteins by biological process. H) Classification of PLM‐miRs proteins by molecular function. I) Western Blot of five key proteins in PLTs, PMVs, LM‐miRs, and PLM‐miRs that mediate the interaction between PLTs and macrophages. J) The glycosylated membrane proteins with correct orientation on PLTs, PMVs, LM‐miRs, and PLM‐miRs were stained with Texas Red‐X‐conjugated WGA (*n* = 3). K) TEM of PLM‐miRs immune‐stained with gold‐conjugated antibody against P‐selectin. L,M) Degradation of miRNA, either in free form or in PLM‐miRs, when exposed to purified RNase (L) or serum‐containing medium (M) for increasing amounts of time. Results are presented as mean ± SD.

To prepare the core, MSNs fully loaded with miRNAs (MSN‐miRs), 3D dendritic biodegradable MSNs were first synthesized by an oil–water bi‐phase stratification approach.^[^ ^31^ ^]^ The spherical dendritic structure of the resulting MSNs (**Figure** **2**A left) was confirmed by transmission electron microscopy (TEM). The size and zeta potential of MSNs measured by dynamic laser scattering (DLS) were 151.40 ± 2.60 nm and −20.43 ± 1.22 mV, respectively (Figure 2B,C). To achieve the payload of miRNA, we adopted a calcium silicate sealing method, which uses Ca^2+^ ions to trap negatively charged miRNAs into the pores of negatively charged bare MSNs.^[^ ^22^ ^]^ The particle size and zeta potential of the resulting MSN‐miRs moderately increased to 156.30 ± 2.83 nm and −15.03 ± 3.34 mV, respectively (Figure 2B,C). The mass loading of miRNA reached ≈26%, as determined by quantification of free miRNA remaining in the supernatant after centrifugation. To fabricate the outer shell PLPs, platelet membrane vesicles (PMVs) were first separated from PLTs by the repeated freeze–thaw method,^[^ ^32^ ^]^ and fusogenic liposomes (LPs) were prepared through thin‐film hydration method according to a previously reported formula.^[^ ^30^ ^]^ Cationic LPs have excellent biomembrane fusion characteristics, so according to the mass ratio of total protein to lipid of 1:500, the obtained negatively charged PMVs and the positively charged LPs are hybridized by the incubation–extrusion method.^[^ ^33^ ^]^ The insertion of platelet membrane will decrease the positive potential of LPs, but the zeta potential of the resulting PLPs can still reach 10.86 ± 1.38 mV, which makes PLPs inherit the fusogenic characteristics of LPs. The coating of positively charged LPs or PLPs on negatively charged MSN‐miRs is also carried out by the incubation–extrusion method.^[^ ^30^ ^]^ The successful encapsulation of MSN‐miRs by LPs (LM‐miRs) or PLPs (PLM‐miRs) was also reflected in changes of particle size and zeta potential (Figure 2B,C). The ultimate morphology of PLM‐miRs is shown in Figure 2A right. Nanoparticles at each stage all have good uniformity (Figure 2A–D). PLM‐miRs can remain relatively stable for at least one week in phosphate buffer saline (PBS) (Figure S1A, Supporting Information), and repeated freezing and thawing will not affect their stability (Figure S1B, Supporting Information).

The retention of membrane proteins on PLM‐miRs was typically determined by sodium dodecyl sulfate polyacrylamide gel electrophoresis (SDS‐PAGE) (Figure 2E). Compared with PMVs, PLM‐miRs retained almost all of the platelet membrane proteins. The protein composition of PLM‐miRs was further investigated by Tandem Mass Tag (TMT) quantitative proteomics analysis. By searching the homo sapiens Uniprot_2020.7.2 database, we identified 469 human membrane proteins in PMVs (Supplementary Appendix), and all these proteins were identified in PLM‐miRs (Supporting Information), indicating that PLM‐miRs inherited the membrane protein species well from PMVs. The expression abundance of proteins related to platelet adhesion and interaction with the immune system are presented in Figure 2F. PLM‐miRs proteins were subsequently classified according to biological process (Figure 2G) and molecular function (Figure 2H). The results showed that PLM‐miRs largely inherited the ability of PLTs to interact with other cells. Additionally, the retention of key proteins, P‐selectin, CD42c, CD47, integrin GP IIb/IIIa, and GP Ib*α*, that mediate the interaction of PLTs and macrophages in PLM‐miRs was further confirmed by Western Blot (Figure 2I).^[^ ^17^ ^]^ To great extent, the presence of these key proteins enables PLM‐miRs to retain the characteristics of platelet that interact with macrophages. To verify the correct orientation of membrane proteins in PLM‐miRs, Texas Red‐X‐conjugated wheat germ agglutinin (WGA) was adopted as a probe to detect glycosylation sites in the extracellular segment of membrane proteins. PLTs, PMVs, and PLM‐miRs, but not LM‐miRs could be stained with WGA (Figure 2J), indicating that the glycoproteins were expressed on the surface of PLM‐miRs with the right orientation. The successful immune staining against P‐selectin on PLM‐miRs, which was observed under TEM, further confirmed the right orientation of proteins in PLM‐miRs (Figure 2K). These results indicate that PLM‐miRs retain the same membrane protein composition and right orientation as PMVs, and therefore has the potential to interact with macrophages like PLTs.

To confirm that PLM‐miRs can protect miRNA from degradation, after incubating PLM‐miRs nanoparticles with RNase or serum‐containing medium for varying times, we verified the integrity of miRNA by agarose electrophoresis (Figure 2L,M). The payload of PLM‐miRs nanoparticles has little degradation; this was in sharp contrast to free miRNA, which was rapidly degraded by RNase either in purified form or within serum. As for release of miRNA from the nanocarrier, Cy5‐labeled PLM‐miRs was incubated with PBS plus 10% fetal bovine serum (FBS) at 37 °C for different time points (Figure S2, Supporting Information). Whereas a minimal amount of release was observed from PLM‐miRs, considerable burst release in the first few hours was observed from naked MSN‐miRs.

### 2.2 Macrophages‐Targeted Assay In Vitro

**Figure 3:**
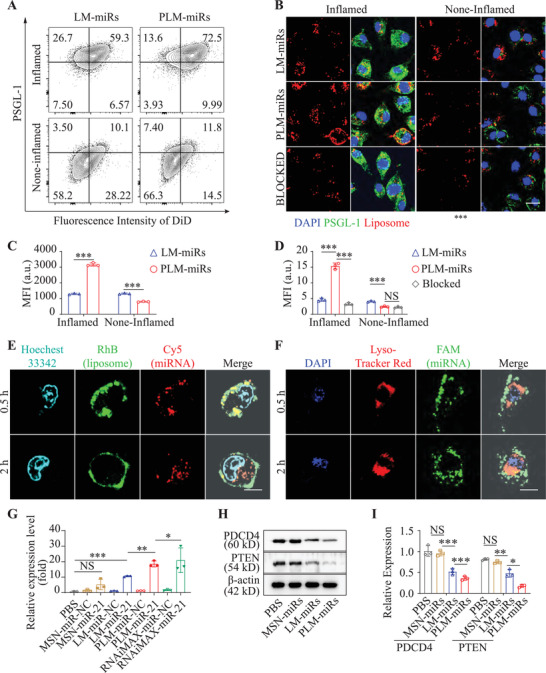
Macrophage targeting ability and cellular internalization of PLM‐miRs in vitro. A) The binding ability of DiD‐labeled LM‐miRs or PLM‐miRs to inflamed (or not) BMDMs was detected by flow cytometry and C) statistically analyzed by FlowJo_V10 software (*n* = 3). B) The binding of LM‐miRs, PLM‐miRs, and anti‐P‐selectin‐blocked PLM‐miRs to inflamed (or not) BMDMs were imaged after staining with PSGL‐1 and D) further quantified by ImageJ (*n* = 3). Red, DiD labeled nanoparticles; Green, PSGL‐1. Scalar bar, 10 µm. E,F) CLSM images of inflamed BMDMs incubated with E) RhB (on liposome, green), Cy5 (on miRNA, red) double labeled PLM‐miRs or F) FAM (on miRNA, green)‐labeled PLM‐miRs for 0.5 and 2 h. Scalar bar, 10 µm. Nucleus were stained with Hoechest 33 342 (cyan) in (E). Lysosomes were stained with Lyso‐Tracker Red (red) and cell nucleus with DAPI (blue) in (F). G) Real‐time RT‐qPCR analysis of the expression of miR‐21 in recipient cells after different treatments (*n* = 3). H) Western blot analysis of the protein levels of PTEN and PDCD4 and was further quantified in (I) by ImageJ (*n* = 3). Results are presented as mean ± SD. ^NS^ *P* > 0.05, *P < 0.05, **P < 0.01, ***P < 0.001.

Focusing on using the biological functions of platelet membrane to achieve targeted delivery of PLM‐miRs, we have explored the binding ability of PLM‐miRs to inflamed (or not) murine bone marrow‐derived macrophages (BMDMs) through flow cytometry (**Figure** **3**A) and immunofluorescence technology (Figure 3B). Both none‐inflamed and inflamed BMDMs were incubated with 1,19‐dioctadecyl‐3,3,39,39‐tetramethylindodicarbocyanine perchlorate (DiD) (on liposome) labeled LM‐miRs or PLM‐miRs for 30 min, respectively. As expected, for inflamed BMDMs, the DiD mean fluorescence intensity (MFI) of PLM‐miRs treated cells calculated from flow cytometry was 2.39‐times that of LM‐miRs treated cells (Figure 3C). For none‐inflamed BMDMs, on the contrary, the MFI of PLM‐miRs group was lower, only 59.66% of the LM‐miRs group (Figure 3C), which may be due to the presence of CD47 on PLM‐miRs. Similar results were also obtained through immunofluorescence staining (Figure 3D). In addition, pre‐blocking with anti‐P‐selectin antibody can significantly reduce the binding of PLM‐miRs to inflamed BMDMs, while the binding of PLM‐miRs to none‐inflamed BMDMs will not be affected (Figure 3D). These results indicated that that PLM‐miRs can achieve selective binding to inflammatory activated macrophages by simulating the interaction between PLTs and macrophages.

### 2.3 Cellular Internalization Pathway of PLM‐miRs

Effective delivery of miRNAs to the cell cytoplasm is a prerequisite for its effectiveness. Therefore, after PLM‐miRs selectively bind to inflamed macrophages, its cellular internalization pathway was studied. To monitor the dynamic process of PLM‐miRs incorporation, the outer lipid envelope of PLM‐miRs loaded with miR‐Cy5 was labeled with rhodamine B (RhB) fluorescence. When cells were incubated with PLM‐miRs for 0.5 h, the fluorescence signals of RhB and Cy5 mainly distributed on the cell surface, and showed obvious co‐localization (Figure 3E). After 2 h incubation, the RhB fluorescence signals were merged to each other, showing the morphology of cell membrane, while Cy5 fluorescence signals of miRNAs were evenly dispersed within the cells (Figure 3E). To further track the fate of cargo miRNAs after entering the cell, we used FAM to label the miRNAs loaded by PLM‐miRs, and Lyso‐Tracker Red to stain the lysosomes of recipient cells. As shown in Figure 3F, after 0.5 and 2 h incubation, the fluorescence signals of miRNA mimics (FAM) and Lyso‐Tracker Red showed negligible colocalization at the both time points. These results indicate that after PLM‐miRs are bound to recipient cells, their content MSN‐miRs are directly released into the cytoplasm through membrane fusion, which effectively avoids the degradation of miRNAs by lysosomal phagocytic system when the nanoplatform enters the cell through endocytosis. After MSN‐miR enters the cell, miRNAs are rapidly released into the cytoplasm with the biodegradation of MSNs, which can be supported by the aforementioned experimental data (Figure S2, Supporting Information).

### 2.4 miR‐21 Delivery Efficiency Assay

To verify the bioactivity of miR‐21 after intracellular delivery, the miR‐21 expression level in recipient inflamed BMDMs was detected by reverse transcription fluorescence quantitative polymerase chain reaction (RT‐qPCR) after six‐hour incubation with PBS, MSN‐miR‐NC, MSN‐miR‐21, LM‐miR‐NC, LM‐miR‐21, PLM‐miR‐NC, and PLM‐miR‐21, respectively. Transfection of miRNAs by using RNAiMAX Reagent was set as a positive control. Except for the RNAiMAX miR‐21 group, the expression level of miR‐21 was highest in the PLM‐miR‐21 group, and it was also significantly up‐regulated in the LM‐miR‐21 group (Figure 3G). In addition, the miR‐21 level in the PLM‐miR‐21 group was obvious higher than that of the LM‐miR‐21 group, but there was no significant difference between the PLM‐miR‐NC and the LM‐miR‐NC group (Figure 3G). These results indicated that the addition of platelet membrane proteins itself will not affect the expression level of miR‐21 in inflamed BMDMs, but it can increase the miR‐21 delivery efficiency of PLM‐miRs. The miR‐21 level was increased slightly but not significantly in MSN‐miR‐21 group due to the deficiency of the membrane fusion ability and targeting ability (Figure 3G). The bioactivity of the delivered miR‐21 was further confirmed by the western blotting analysis of two representative downstream targets, phosphatase and tensin homologue (PTEN) and programmed cell death protein (PDCD4) (Figure 3H). The results showed that both PLM‐miRs and LM‐miRs can effectively down‐regulate the expression levels of the above two target proteins, especially PLM‐miRs performed best (Figure 3I).

### 2.5 PLM‐miRs Reprogram Inflamed BMDM to M2 Phenotype In Vitro

**Figure 4:**
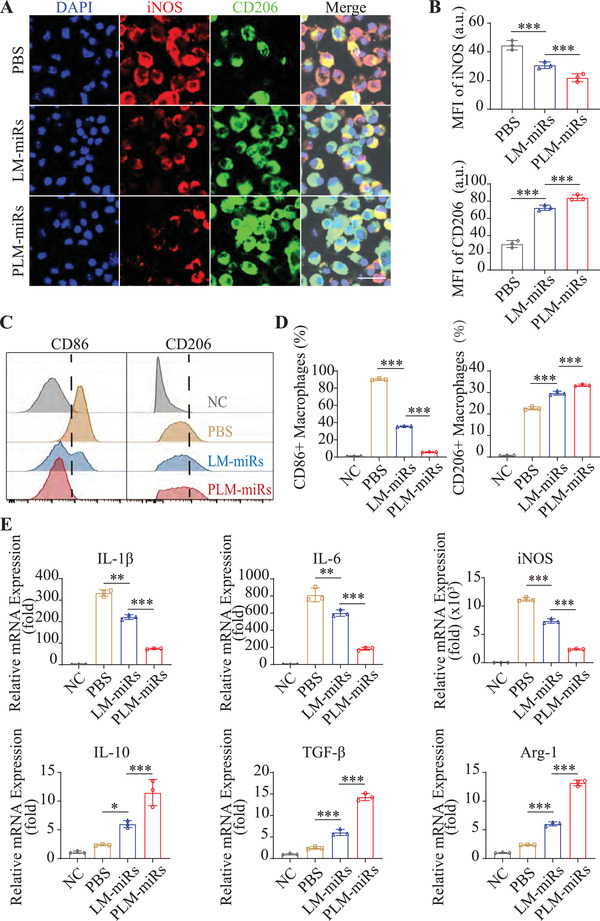
PLM‐miRs reprogram inflamed BMDM to M2 phenotype in vitro. A) CLSM images indicating phenotypes of inflamed BMDMs after treated by PBS, LM‐miRs or PLM‐miRs. Scalar bar, 20 µm. B) MFI quantification of iNOS (M1) and CD206 (M2) in (A) (*n* = 3). C) Flow cytometry assay and D) statistical analyze of the polarization of inflamed BMDMs after treated by PBS, LM‐miRs, or PLM‐miRs (*n* = 3). M1 and M2 subtypes were indicated by CD86 and CD206, respectively. E) Real‐time RT‐qPCR analysis of the expression levels of M1 markers (IL‐1*β*, IL‐6, iNOS) and M2 markers (IL‐10, TGF‐*β*, Arg‐1) in inflamed BMDMs after treated by PBS, LM‐miRs, or PLM‐miRs (*n* = 3). Results are presented as mean ± SD. ^NS^ *P* > 0.05, *P < 0.05, **P < 0.01, ***P < 0.001.

To confirm the effect of PLM‐miRs on the polarization state of macrophages, PBS, LM‐miRs, or PLM‐miRs were added to BMDMs after inflammatory activation, and unstimulated macrophages were served as a control. We then detected BMDMs phenotype distribution in each group. Analysis by immunofluorescence staining indicated that the proportion of the M1 subpopulation was obviously reduced, while the proportion of the M2 subpopulation increased in the LM‐miRs and PLM‐miRs groups (**Figure** **4**A,B). Meanwhile, the regulatory effect of PLM‐miRs was greater than that of LM‐miRs (Figure 4B), which may be attributed to its targeting ability. The flow cytometry analysis obtained similar results as immunofluorescence staining (Figure 4C,D). In addition, the expression levels of M1 and M2 marker genes were semi‐quantitatively analyzed at the transcriptional level by real time RT‐qPCR. The results showed that both PLM‐miRs and LM‐miRs significantly down‐regulated the expression of M1 marker genes (interleukin 1*β* (IL‐1*β*), IL‐6, and inducible nitric oxide synthase (iNOS)), while up‐regulated the expression of M2 marker genes (transforming growth factor *β* (TGF‐*β*), IL‐10, and Arginase 1 (Arg‐1)), and PLM‐miRs performed better (Figure 4E). These results revealed that PLM‐miRs can effectively reprogram inflammatory macrophages toward a reparative phenotype in vitro.

### 2.6 Biodistribution and Targeting Specificity of PLM‐miRs In Vivo

**Figure 5:**
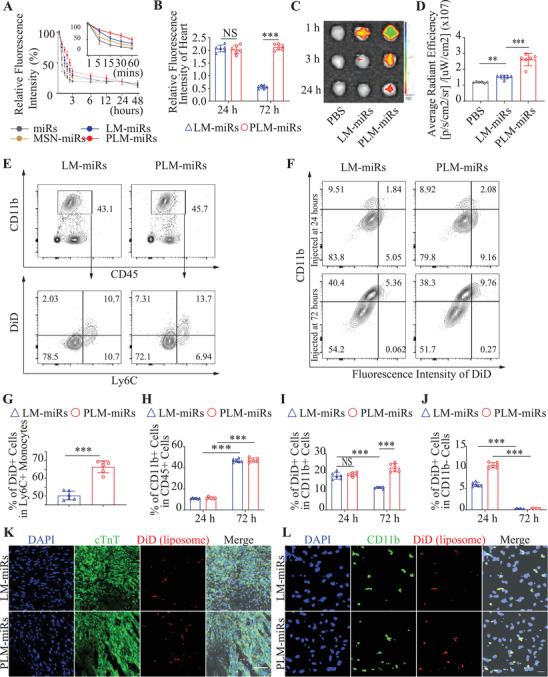
Biodistribution and targeting specificity of PLM‐miRs in vivo. A) Circulation profiles of miRs, MSN‐miRs, LM‐miRs, and PLM‐miRs in healthy mice after intravenous injection (*n* = 6). B) RFI of heart tissue homogenate after treated with DiD (on liposome) labeled LM‐miRs or PLM‐miRs at 24 or 48 h post injury (*n* = 6). C) IVIS images of three timepoints (1, 3, 24 h) of LM‐miRs or PLM‐miRs accumulated in the hearts after i.v. injected in MI/R mice and D) were further quantified the data of the first time point (*n* = 6). E) Flow cytometry analysis of the binding ability of PLM‐miRs to Ly6C+ monocytes in the blood circulation after intravenous injection at 72 h post‐MI and G) was further quantified (*n* = 6). F) Flow cytometry and statistical analysis of H) CD11b+, I) CD11b+DiD+, and J) CD11b+DiD‐ cells isolated from MI/R induced murine hearts after exposure to either LM‐miRs or PLM‐miRs injected at 24 or 72 h post‐MI (*n* = 6). K) CLSM images of heart sections showing the accumulation of LM‐miRs and PLM‐miRs after immuno‐stained with cardiac troponin T (cTnT). Red, DiD (on liposome) labeled nanoparticles; Green, TnT. Scalar bar, 50 µm. L) CLSM images of heart sections showing the colocalization between monocytes/macrophages and LM‐miRs or PLM‐miRs after immuno‐stained with CD11b. Red, DiD (on liposome) labeled nanoparticles; Green, CD11b. Scalar bar, 20 µm. Results are presented as mean ± SD. ^NS^ *P* > 0.05, *P < 0.05, **P < 0.01, ***P < 0.001.

To systematically evaluate the in vivo performance of PLM‐miRs, the blood circulation profile was investigated first. Cy5 (on miRNA) labeled miRs, MSN‐miRs, LM‐miRs, or PLM‐miRs were injected into healthy mice through tail vein injection. As shown in **Figure** **5**A, both naked miRs and MSN‐miRs are quickly eliminated from the blood. The encapsulation of PEGylated liposomes significantly increased the blood circulation time of LM‐miRs. On this basis, the presence of platelet membrane components further extends the biological half‐life of PLM‐miRs (Figure 5A). To further explore the in vivo biodistribution of DiD (on liposome) labeled LM‐miRs and PLM‐miRs after administered through tail vein injection to MI/R induced mice, the fluorescence intensity (FI) of tissue homogenates of main organs was detected. There was no difference in the accumulation of LM‐miRs and PLM‐miRs in the heart when injected at 24 h post‐reperfusion (Figure 5B). While the FI of heart homogenates in PLM‐miRs group valued 3.90 times that of LM‐miRs group (Figure 5B). The decrease in the accumulation of LM‐miRs coincides with the disappearance of the EPR effect. In addition, compared with LM‐miRs, the uptake of PLM‐miRs injected at the both time points by liver and spleen was reduced (Figure S3A,B, Supporting Information), This might be attributed to the fact that monocytes and macrophages in the liver and spleen are mainly in the resting state, and the presence of CD47 reduces the uptake of PLM‐miRs by monocytes/macrophages. In vivo spectrum imaging system (IVIS) were also used to detect the distribution of DiD (on liposome) labeled LM‐miRs or PLM‐miRs in the major organs of MI/R induced mice after administered 72 h post‐MI (Figure 5C and Figure S3C, Supporting Information). The results showed that the accumulation of PLM‐miRs in the injured heart was much higher than all other groups (Figure 5D), and PLM‐miRs were captured less by liver and spleen than LM‐miRs (Figure S3D, Supporting Information), which is consistent with the aforementioned results. There was no significant difference in their distribution in other major organs (Figure S3D, Supporting Information).

To further explore the underlying mechanism of the selective accumulation of PLM‐miRs in the injured heart, we first used flow cytometry to verify the ability of DiD (on liposome) labeled PLM‐miRs to target Ly6C+ monocytes in mice blood circulation after tail vein injection at 72 h post‐MI/R (Figure 5E). The proportion of DiD+Ly6C+ cells in PLM‐miRs group was significantly higher than that of LM‐miRs group (Figure 5G), indicating that the presence of platelet membrane components effectively enhanced the binding of PLM‐miRs and Ly6C+ monocytes. To verify whether PLM‐miRs can reach the heart injury area through the carrying of monocytes, we used flow cytometry to analyze the aggregation of DiD (on liposome) labeled LM‐miRs or PLM‐miRs in the monocytes of cardiac tissue (Figure 5F). At 24 and 72 h post‐reperfusion, the proportion of CD11b+ cells in white blood cells (CD45+ cells) was 11.27% and 46.38%, respectively (Figure 5H), which is consistent with the previously reported dynamics of inflammatory cells after MI.^[^ ^6^, ^18^ ^]^ At 24 h post‐reperfusion, the aggregation of PLM‐miRs in CD11b+ cells were slightly higher than that of LM‐miRs, but there was no statistical difference (Figure 5I). As expected, the proportion of DiD+ cells in CD11b+ cells of the LM‐miRs group dropped from 18.02 to 12.09% after administered at 72 h post‐MI/R, while the proportion in the PLM‐miRs group instead rose from 18.82 to 23.16% (Figure 5I). It is worth noting that, compared with 24 h post‐reperfusion, administration at 72 h after reperfusion will sharply decrease the aggregation of PLM‐miRs and LM‐miRs in CD11b‐ cells (Figure 5J). This phenomenon may be due to the disappearance of the EPR effect that caused the nanoparticles cannot reach the local area and interact with CD11b‐ cells. Immunofluorescence staining of frozen sections further confirmed the targeting ability of DiD (on liposome) labeled PLM‐miRs to the injured area of mice heart and revealed that PLM‐miRs mainly distributed in the border zone (Figure 5K and Figure S3E). In addition, compared to LM‐miRs, the more obvious co‐localization of DiD (on liposome) labeled PLM‐miRs and CD11b+ cells in injured heart (Figure 5L and Figure S3F,G, Supporting Information) can be another solid evidence proving that PLM‐miRs can be carried by monocytes to the injured area of MI/R induced heart. In summary, these results indicate that PLM‐miRs have considerable circulating time in the blood circulation of mice, and it can bind to monocytes that are chemotaxis toward the injured heart, and then be carried to the injured area.

### 2.7 PLM‐miRs Promote Reparative Polarization of Macrophages In Vivo

**Figure 6:**
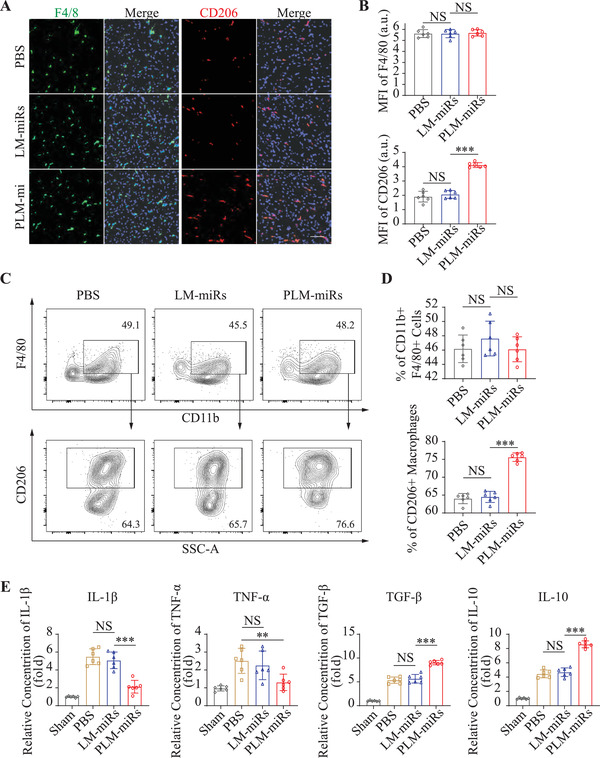
PLM‐miRs promote reparative polarization of macrophages in vivo. A) CLSM images of MI/R injured heart sections showing the total (F4/80+) and M2 subtype (CD206+) macrophages after treated by PBS, LM‐miRs, or PLM‐miRs. Scalar bar, 50 µm. B) MFI quantification of F4/80 (total) and CD206 (M2) in (A) (*n* = 6). C) Flow cytometry assay and D) statistical analysis of M2 subtype (CD206+) macrophages isolated from MI/R induced murine hearts after treated by PBS, LM‐miRs, or PLM‐miRs (*n* = 6). E) ELISA analysis of IL‐1*β*, TNF‐*α*, TGF‐*β*, and IL‐10 concentrations in heart homogenate of MI/R induced mice after treated by PBS, LM‐miRs, or PLM‐miRs (*n* = 6). Results are presented as mean ± SD. ^NS^ *P* > 0.05, *P < 0.05, **P < 0.01, ***P < 0.001.

After being carried by monocytes to the injured area of the heart, the effect of PLM‐miRs on the phenotype of the successive macrophages was evaluated by immunofluorescence staining (**Figure** **6**A). When the total amount of macrophages (F4/80+ cells) remained unchanged, the proportion of M2 subtype macrophages (CD206 + cells) in the PLM‐miRs group increased significantly (Figure 6B). Flow cytometry also confirmed this result (Figure 6C). The results suggested that PLM‐miRs can significantly increase the proportion of M2 subtypes of cardiac macrophages, while LM‐miRs has little effect, compared with the control (Figure 6D). Furthermore, enzyme linked immunosorbent assay (ELISA) analysis of the concentration of inflammatory cytokines (IL‐1*β*, tumor necrosis factor *α* (TNF‐*α*), TGF‐*β*, and IL‐10) in heart homogenate of MI/R induced mice were adopted to verify the immune‐regulatory efficiency of PLM‐miRs. The results showed that PLM‐miRs can significantly reduce IL‐1*β* and TNF‐*α* concentration, while obviously increasing TGF‐*β* and IL‐10 concentration (Figure 6E). Overall, these results have powerfully demonstrated that PLM‐miRs could effectively promote the reparative polarization of inflamed macrophages. The difference in the performance of LM‐miRs in vivo and in vitro may be attributed to the fact that membrane fusion properties make it effective in cell experiments, while the lack of platelet membrane components prevents it from effectively binding to monocytes in vivo.

### 2.8 Cardiac Protection Efficiency of PLM‐miRs

**Figure 7:**
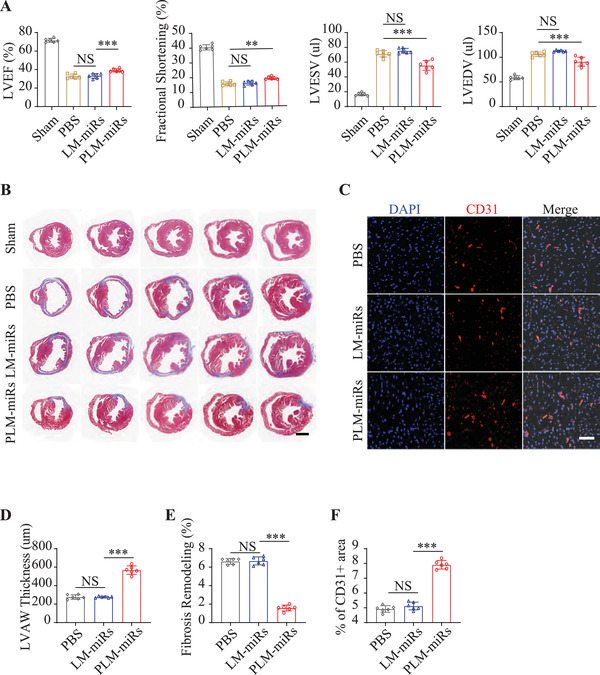
Cardiac protection efficiency of PLM‐miRs. A) Cardiac function was assessed by echocardiography at 4 weeks after treatment (*n* = 6). B) Masson staining of MI/R heart paraffin sections at 4 weeks after various treatments. Scalar bar, 1 mm. C) CLSM images of CD31+ signals in MI/R injured murine heart sections after PBS, LM‐miRs, or PLM‐miRs administered (*n* = 6). Scalar bar, 50 µm. D) LVAW thickness and E) fibrosis remodeling in (B) was quantified by using ImageJ software (*n* = 6). F) Quantification of percentage of CD31+ area in MI/R injured murine heart sections after PBS, LM‐miRs, or PLM‐miRs administered (*n* = 6). Results are presented as mean ± SD. ^NS^ *P* > 0.05, *P < 0.05, **P < 0.01, ***P < 0.001.

To verify the cardiac protection of PLM‐miRs, cardiac function of MI/R induced mice was evaluated by echocardiography at 4 weeks after PBS, LM‐miR or PLM‐miRs administered, respectively (**Figure** **7**A). Compared with other groups, the left ventricular ejection fraction (LVEF) of the PLM‐miRs group was preserved to the greatest extent (50.07% vs 33.75% vs 31.61%, PLM‐miRs vs LM‐miRs vs Ctrl) (Figure 7A). The fractional shortening analysis has confirmed that PLM‐miRs can effectively preserve the contraction efficiency of cardiomyocytes, while LM‐miRs show a negligible effect (Figure 7A). Statistical analysis of left ventricular end‐systolic volume (LVESV) and left ventricular end‐diastolic volume (LVEDV) suggested that PLM‐miRs can effectively prevent the deterioration of mouse cardiac function post‐MI/R (Figure 7A). After that, to assess the cardiac remodeling, the heart paraffin sections of different layers were used to quantify the preserved left ventricular anterior wall (LVAW) thickness and fibrosis remodeling through Masson staining (Figure 7B). As expected, the PLM‐miRs group has preserved the most myocardium and the subsequent fibrosis is the lightest, compared with control groups (Figure 7D,E). To explore whether the regulation of the polarization state of macrophages by PLM‐miRs contributes to the maturation of sprouting vessels, immunofluorescence staining against CD31, a typical mature endothelial marker, were conducted at 7 d post‐MI/R (the 5th day after treatment) (Figure 7C). The PLM‐miRs group possess the most microvascular density (percentage of CD31+ area) compared with control, suggesting the best pro‐angiogenesis effect (Figure 7F). These results indicated that PLM‐miRs can promote the maturation of newly blood vessels by regulating the polarization state of macrophages, thereby effectively improving MI/R‐induced cardiac function in mice.

### 2.9 Biosafety Assessment of PLM‐miRs

The toxicity of PLM‐miRs to cells in vitro and its biological safety to mice in vivo were under our consideration. In vitro, CCK8 assay revealed that PLM‐miRs has no obvious cytotoxicity to BMDMs and rat embryonic cardiomyocyte (H9C2) (Figure S4A, Supporting Information). In vivo, after PBS or PLM‐miRs were injected into healthy mice through tail vein injection, a series of safety‐related tests were carried out. Regarding the immune response, the serum levels of IL‐1*β* and TNF‐*α* was quantified by ELISA assay on the 3rd day after PLM‐miRs injection. The concentration of IL‐1*β* and TNF‐*α* in the serum of PLM‐miRs treated mice was not significantly different from that in the control group (Figure S4B,C, Supporting Information), indicating that PLM‐miRs injection will not cause acute inflammation. The serum levels of general antibodies, immunoglobulin G (IgG) and IgM, were also quantified by ELISA assay, and no statistical difference was observed between group PLM‐miRs and control, indicating that there was no potential immune response after PLM‐miRs administration (Figure S4D,E, Supporting Information). With respect to organ toxicity, biochemical test of liver and renal functions was conducted. The results showed that PLM‐miRs had no adverse effects on liver and renal function (Figure S4F,G, Supporting Information). Meanwhile, compared with control, no histopathological changes were observed in major organs of healthy mice treated with PLM‐miRs (Figure S4I, Supporting Information). The application of PLTs biomimetic nanoparticles needs the consideration of its impact on clotting function. Compared with the control group, normal activated partial thromboplastin time (APTT), prothrombin (PT), and fibrinogen levels (Fbg) suggest that PLM‐miRs have no abnormal effects on the coagulation and fibrinolytic system in vivo (Figure S4H, Supporting Information). All these results demonstrated that the biological safety of PLM‐miRs was up to standard, which is essential for its potential clinical application in the later stage.

## 3 Discussion and Conclusion

In this study, the platelet‐like fusogenic nano‐delivery platform we developed realized the active targeted delivery of miR‐21 to monocytes/macrophages in the injured area of MI/R mice heart. Our data indicate that i) PLM‐miRs can efficiently load miR‐21 and protect it from degradation by RNase. ii) In the blood circulation of MI/R induced mice, PLM‐miRs can bind to Ly6C+ monocytes that are chemotaxis to the injured heart, and then be carried to the injured area to achieve active targeting of inflammatory monocytes, and macrophages that evolved from them. iii) PLM‐miRs can effectively deliver miR‐21 into the cytoplasm of monocytes/macrophages through membrane fusion, thereby achieving anti‐inflammatory reprogramming of inflammatory macrophages. The prominent advantage of PLM‐miRs is that it organically integrates the targeting initiative of platelet membrane biomimetic technology, the delivery efficiency of cationic liposomes and the high loading efficiency of MSN for miRNA.

The main innovation of this study is that we use cationic liposomes as the backbone of PLPs. Most of the previously reported platelet mimicry studies used platelet membranes directly for coating or used anionic liposomes for chimerization.^[^ ^34^, ^35^, ^36^ ^]^ However, for a miRNA therapy, a sufficiently high content delivery efficiency is as important as precise targeting. Cationic liposomes can directly deliver their contents to the cytoplasm of recipient cells through membrane fusion, which may be more conducive to achieving a higher concentration of miRNA in target cells.^[^ ^30^ ^]^ Therefore, we tried to inlay the platelet membrane with cationic liposomes to give it membrane fusion characteristics, thereby increasing the content delivery efficiency of platelet‐coated nanoparticles. The successful chimerization of platelet membrane and cationic liposome was verified in this study. First, the outer shell of PLM‐miRs contains the same protein composition and correct protein orientation as the platelet membrane. Second, PLM‐miRs can effectively mimic PLTs to target monocytes/macrophages both in vivo and in vitro. Finally, PLM‐miRs can efficiently deliver miR‐21 into the cytoplasm of recipient cells by means of membrane fusion, thereby achieving reprogramming of inflammatory macrophages.

Our original intention of using platelet membrane as the compass of PLM‐miRs is to actively target monocytes/macrophages in a way that does not rely on the EPR effect, so that the formula can be administered when the peak of macrophage recruitment comes. Therefore, in the design of the entire study, we focused on verifying the specific pathways for PLM‐miRs to reach the target area. Our results support that the embedding of platelet membranes can help PLM‐miRs reach the injured area of the mice heart by riding Ly6C+ monocytes in the blood circulation.

It is worth noting that a research team has successfully achieved the intervention of the polarization state of macrophages post‐MI/R within an ideal time window by intramyocardial injection.^[^ ^14^ ^]^ The PLM‐miRs we developed are injected through the tail vein, which greatly avoids invasive operations and has more clinical transformation potential. Further refinements are expected to help our formula become a promising approach of immunotherapies.

## 4 Experimental Section

### Materials

Hexadecyl trimethyl ammonium bromide (CTAB), triethanolamine (TEA), 0.6 wt% ammonium nitrate (NH_4_NO_3_), and cyclohexane were purchased from Sigma‐Aldrich (USA). Tetraethyl orthosilicate (TEOS) was purchased from Aladdin Reagent Co., Ltd. Calcium dichloride (CaCl_2_) were purchased from Sangon Biotech (Shanghai) Co., Ltd. 1,2‐Dimyristoyl‐sn‐glycero‐3‐phosphocholine (DMPC), methoxy poly (ethylene glycol)‐1,2‐distearoyl‐sn‐glycero‐3‐phosphoethanolamine‐N (mPEG200‐DSPE), and 1,2‐dioleoyl‐3‐trimethylammoniumpropane (DOTAP) were purchased from Corden Pharma (Switzerland). Lissamine rhodamine B (RhB) and DiD was purchased from Thermo Fisher Scientific (USA).

### Preparation of PLM‐miRs

MSNs were synthesized by a previously reported procedure.^[^ ^31^ ^]^ Briefly, 3 grams of CTAB and 0.15 mL of TEA were added to 60 mL of distilled water and stirred gently at 60 °C for 1 h. Then, 4 mL of TEOS and 16 mL of cyclohexane (5 v/v%) were carefully added to the water‐CTAB‐TEA solution and kept at 60 °C in an oil magnetics stirrer for 24 h. The products were collected by centrifugation and washed with ethanol three times. The resulting MSNs were resuspended with 0.6 wt% NH_4_NO_3_ in ethanol and stirred at 60 °C for 3 h thrice to remove the template. The final products were washed with ethanol and water three times and lyophilized in distilled water at a concentration of 20 mg mL^−1^.

The load of miR‐21 mimics (Gene Pharma, China) by MSNs was achieved by a reported calcium silicate sealing method.^[^ ^22^ ^]^ Briefly, 150 µL miRNA mimics (20 um in deionized water) was pipetted gently with 100 µL MSNs (10 mg mL^−1^ in deionized water) and added to 750 µL 4 m CaCl2 stock solution. The mixture was agitated for 60 min and purified by successive dispersion in/centrifugation from RNAse free deionized water, 70% ethanol, and 100% ethanol in sequence. The loading of miRNA negative control labeled with Cy5 or FAM (Gene Pharma, China) was also achieved through the same method. To verify miRNA loading efficiency, supernatants and pellets from each centrifugation step were collected and assayed for ultraviolet absorption using a NanoDrop spectrophotometer (Denovix DS‐11, USA).

The synthesis of PLPs mainly includes the following three steps: 1) fibrication of PMVs; 2) preparation of fusogenic liposomes; 3) hybridization of PMVs and LPs. Briefly, the PLTs were collected from platelet rich plasma and platelet membrane fractions were derived by a −80 °C repeated freeze–thaw process as described previously.^[^ ^32^ ^]^ To obtain PMVs, platelet membrane fractions were successively extruded 20 times through 1.0, 0.4, and 0.2 um polycarbonate membrane (Nuclepore Track‐Etched Membranes, Whatman, UK), using a LiposoFast extruder apparatus (Avestin, Canada). After centrifugation at 5000 g for 15 min, the obtained PMVs were resuspended in PBS, and the protein concentration was quantified using BCA assay kit (Beyotime, China). LPs were prepared by the thin‐film hydration method.^[^ ^30^ ^]^ Briefly, DMPC, mPEG2000‐DSPE, and DOTAP were dissolved in trihalomethane at the molar ratio of 76.2:3.8:20. The lipid film was prepared by evaporating the organic solvent in a 37 °C water bath under vacuum. DiD or RhB labeled lipid films were obtained by adding DiD or RhB into the initial solution at the v/v ratio of 1:1000. The evaporated dried film was hydrated with 1× PBS (pH 7.4) and incubated at 37 °C for 30 min, mixed vigorously and further sonicated to obtain a clear suspension of lipid. After the lipid suspension was extruded through the same series of membrane as above, LPs with uniform particle size were obtained. The obtained PMVs and LPs were hybridized with a mass ratio of total protein: lipid of 1:500 through an incubation–extrusion method.^[^ ^33^ ^]^ Briefly, PMVs were first incubated with LPs in an ultrasonic bath at RT for 20 min, and then successively extruded 20 times through 1.0, 0.4, and 0.2 um polycarbonate membrane.

Finally, the MSN‐miRs were coated with LPs or PLPs by the same incubation–extrusion method. The resulting particles were washed three times at each step by centrifugation in Microcon‐30 kD Centrifugal Filter Unit (EMD Millipore) spinning at 5000 × g at RT to remove uncoated LPs or PLPs. LM‐miRs or PLM‐miRs were stored at 4 °C until use.

### Characterization of PLM‐miRs

The morphology and size of nanoparticles at each stage were observed under a TEM (Tecnai G2 Spirits Twin, FEI, USA) after negative staining with 1% phosphotungstic acid. The size distribution and zeta potential were measured by a DLS detector (Zetasizer‐ZS90, Malvern Instruments, UK). After storing in PBS for different time points (1, 3, 5, 7 days), or after 7 freeze–thaw cycles, we measured the particle size of PLM‐miRs with DLS to detect its in vitro stability.

To verify the protein composition of the nanoparticles made by this method, quantitative proteomics analysis of PLM‐miRs was conducted by TMT technology, and the proteome of PLM‐miRs was compared with that of PMVs. TMT quantitative proteomics analysis mainly includes the following 6 steps: total protein extraction, protein quality test, TMT labeling of peptides, separation of fractions, liquid chromatography−tandem mass spectrometry (LC‐MS/MS) analysis and data analysis. Details are included in Supplementary Materials. Typical proteins that mediate the interaction between PLTs and macrophages on PLM‐miRs were also verified by western blot. Briefly, samples were all separated on 10% SDS polyacrylamide gels (BioRad, US), then transferred to polyvinylidene fluoride membrane, and then probed with antibodies specific for GP IIb/IIIa (sc‐21783, Santa Cruz, USA), P‐selectin (sc‐8419, Santa Cruz, USA), GP Ib*α* (4067‐GP‐050, R & D Systems, USA), CD42c (ab96565, Abcam, Japan) and CD47 (ab175388, Abcam, Japan). After stained with horseradish peroxidase‐conjugated secondary antibodies (Biotech Well, China) with corresponding species reactivity, proteins were imaged using a Bio‐Rad Chemi Doc imaging system.

In view of WGA could selectively bind the N‐acetylneuraminic acid residues and N‐acetylglucosamine of glycoproteins, fluorescent WGA was used as a probe verify the right orientation of the membrane proteins on the PLM‐miRs.^[^ ^37^ ^]^ Briefly, samples (PLTs, PMVs, LM‐miRs, or PLM‐miRs) were incubated with 10 µg mL^−1^ of Texas Red‐X‐conjugated WGA (excitation/emission = 595/615 nm, Thermo Fisher Scientific, USA) in PBS for 30 min and then washed through dialysis. The FI of each sample was then measured using a Multiplate Reader (Molecular Devices, USA). TEM technology was also used to detect the orientation of the proteins on the surface of PLM‐miRs. After sequentially stained with Mouse‐anti‐P‐selectin primary antibody (sc‐8419, Santa Cruz) and gold conjugated secondary antibody (G7652‐4ML, Sigma, USA), PLM‐miRs were observed under TEM.

For the miRNA degradation study in purified RNase, a working solution of an RNase cocktail enzyme mix (Invitrogen, USA) was prepared by diluting 1:1000 with distilled water. Then, 10 µL of the diluted enzyme mix was added into 100 µL aliquots containing 50 pmol of miRNA in free form or in PLM‐miR, and the mixtures were incubated at 37 °C for increasing amounts of time. Each sample was then prepared with DNA loading buffer (Beyotime, China) and loaded into a 1.5% agarose gel (BIOWEST, France) containing Gel Red nucleic acid stain (Beyotime, China). The agarose gel was run in 1 × tris‐acetate‐EDTA buffer (Sangon Biotech, China) at 120 V for 30 min and imaged using a Bio‐Rad Chemi Doc imaging system. For the miRNA degradation study in serum‐containing medium, 100 µL of Dulbecco's modified Eagle's medium (DMEM, Gibco, USA) containing 10% FBS was added into 10 µL aliquots containing 50 pmol of miRNA in free form or in PLM‐miRs, and the mixtures were incubated at 37 °C for increasing amounts of time. Samples were then prepared and run as described above.

To quantify miRNA release, nanoparticles were resuspended in PBS at 37 °C plus pH 7.4. At predetermined time points, aliquots from each group were centrifuged to pellet the nanoparticles, and the fluorescence of the FAM dye (excitation/emission = 494/520 nm) in the supernatant was measured using a multiplate reader.

### Cell Culture

BMDMs were extracted from 6–8w C57BL/6 mice according to previously reported protocol.^[^ ^38^ ^]^ Briefly, after mice were euthanized by rapid cervical dislocation, their femurs and tibias were quickly separated, and then the bone marrow cavity was flushed with sterile PBS through a 7 µm cell strainer to obtain a single cell suspension. The isolated bone marrow cells were centrifuged and resuspended in DMEN containing 10% FBS, 1% penicillin/streptomycin (P/S) and 20 ng mL^−1^ recombination murine Macrophage Colony Stimulating Factor (M‐CSF, PeproTech, USA) for 7 days to obtain mature macrophages. The successful extraction of BMDMs was confirmed by flow cytometry. Pro‐inflammatory M1 activation was achieved by stimulating mature BMDMs with 100 ng mL^−1^ lipopolysaccharide (Sigma, USA) and 20 ng mL Interferon‐Gamma (IFN‐*γ*, PeproTech, USA) for 24 h. The pro‐regenerative M2 phenotype was achieved by inducing BMDMs with 20 ng mL^−1^ IL‐4 (PeproTech, USA) and 20 ng mL^−1^ IL‐13 (PeproTech, USA) for 24 h. H9C2 (ATCC, USA) was cultured in DMEM containing 10% FBS and 1% P/S. Cells were all cultured in a humidified cell culture incubator (ThermoFisher Scientific, USA) at 37 °C with 5% carbon dioxide (CO_2_).

### Macrophages‐Targeted Assay In Vitro

First, BMDMs were pro‐inflammatory activated by the previously described method and none‐inflamed BMDMs served as a control. Inflamed and none‐inflamed BMDMs were all incubated with PBS, DiD (on liposome) labeled LM‐miRs or PLM‐miRs, respectively, for 30 min. The interaction between nanoparticles and cells was analyzed by immunofluorescence assay and flow cytometry. For immunofluorescence assay, the obtained adherent cells were washed three times with PBS, then fixed with 4% paraformaldehyde (Beyotime, China) for 20 min, and then blocked with 3% BSA in PBS 1 h. The resulting cells were incubated with primary antibody Rat‐anti‐PSGL‐1 (sc‐53514, Santa Cruz, USA) overnight at 4 °C. After washing three times with PBS, the samples were stained with the secondary antibody Alexa Fluor 488‐anti‐Rat (ab175473, Abcam, Japan). DAPI (Beyotime, China) was used as a nuclei indicator. Fluorescence signals were detected by a confocal laser scanning microscope (CLSM, Olympus, Japan). For flow cytometry, cells were collected by centrifugation at 1000 rpm for 5 min after being digested by 0.25% trypsin (Gibco, USA). Resuspend 1–2 × 10^6^ cells per 100 µL Staining Buffer (Well Biotech, China) as a test for subsequent fluorescent antibody staining at RT for 40 min. Unbound antibodies were removed by centrifugation, and all samples were tested by BD FACS Aria III (BD biosciences, USA) within 1 h. To more accurately quantify the number of PLM‐miRs bound to each group of cells, we used FlowJo_V10 software to quantify the MFI of the DiD fluorescence signal in each group of cells. Specific antibodies mainly include PE‐anti‐F4/80 (#565 410, eBioscience, USA) and Alexa Flour 647‐anti‐PSGL‐1 (#51‐1621‐82, eBioscience, USA).

### Cellular Internalization of PLM‐miRs

After pro‐inflammatory activation of BMDMs, 200 µL of PLM‐miRs was added to the culture medium and removed at different time points (30 min; 2 h). DAPI and Lyso‐Tracker Red (Beyotime, China) were co‐stained to explore whether FAM (on miRNA) labeled PLM‐miRs enter the cell via endocytosis. The cellular internalization of PLM‐miRs double labeled by Cy5 (on miRNA) and RhB (on liposome) was also under the dynamic monitoring of CLSM, and Hoechest 33 342 (Beyotime, China) served as a nuclear indicator.

### miRNA Delivery Efficiency Assay

BMDMs were extracted and seeded in 6‐well plates. Pro‐inflammatory activated macrophages were treated with PBS, MSN‐miR‐NC, MSN‐miR‐21, LM‐miR‐NC, LM‐miR‐21, PLM‐miR‐NC, PLM‐miR‐21, respectively, for 24 h. Transfection of miR‐NC or miR‐21 by Lipofectamine RNAiMAX Reagent (Invitrogen, USA) to pro‐inflammatory activated macrophages was also adopted as positive controls. The expression level of miR‐21 was determined by real‐time RT‐qPCR. Total RNA was extracted from all samples by using Trizol reagent (Invitrogen, USA) according to the manufacturer's protocol and quantified by NanoDrop in triplicate. To extract short chain RNA as completely as possible, the chloroform with extracted RNA was frozen at −80 °C for 2 extra hours after adding isopropanol. 500 ng of total RNA was reverse‐transcribed into complementary DNA (cDNA) with a miRcute Plus First‐Strand cDNA Kit (TIANGEN BIOTECH, China). Reverse‐transcription was performed by 60 min of incubation at 42 °C followed by 95 °C for 3 min, 4 °C for ∞ with the T100^ ^Thermal Cycler (Bio‐Rad, USA). RT‐qPCR was performed using cDNA with the miRcute Plus miRNA qPCR Kit (TIANGEN BIOTECH, China) and the poly‐A miRNA RT‐qPCR primer set for miR‐21 and U6 (TsingKe, China). RT‐qPCR was performed by 15 min of incubation at 95 °C followed by 40 cycles of 94 °C for 20 s, 60 °C for 34 s with the CFX96TM Real‐time RT‐PCR System (Bio‐Rad, USA). U6 was adopted as the internal control for the quantitation of miR‐21. Primer sequences are as follows: miR‐21 forward: 5′‐GCTAGCTTATCAGACTGATGTTG‐3′; U6 forward: 5′‐CTCGCTTCGGCAGCACA‐3′. As target molecule of miR‐21, the expression of PDCD4 and PTEN were also detected by Western Blot. Rabbit‐anti‐PDCD4 antibody (#9535T) and Rabbit‐anti‐PTEN antibody (#9188T) were purchased from Cell Signaling Technology. The relevant steps are the same as mentioned above.

### Polarization State Analysis of Macrophages In Vitro

BMDMs were seeded in 6‐well plates, then pro‐inflammatory activated, and then treated with PBS, LM‐miRs, PLM‐miRs, respectively, for 24 h. The none‐inflamed BMDMs were also used as a control. To verify the effect of our nanoparticles on the polarization state of macrophages in vitro, we used RT‐qPCR to detect the mRNA expression levels of M1 markers (IL‐1*β*, IL‐6, and iNOS) and M2 markers (IL‐10, TGF‐*β*, and Arg‐1). The RNA extraction method was the same as previously described, except for the extra 2 h of −80 °C freezing. PrimeScript RT Master Mix (TaKaRa, Japan) and TB Green Premix Ex Taq (TaKaRa, Japan) were used in RT‐qPCR. The reverse‐transcription was achieved by 15 min of incubation at 37 °C followed by 87 °C for 5 s, 4 °C for ∞ with the T100^ ^Thermal Cycler (Bio‐Rad, USA). RT‐qPCR was performed by 30 s of incubation at 95 °C followed by 40 cycles of 95 °C for 3 s, 60 °C for 30 s with the CFX96TM Real‐time RT‐PCR System (Bio‐Rad, USA). The primer sequences used are as follows: *β*‐actin forward: 5′‐GTGACGTTGAC ATCCGTAAAGA‐3′, *β*‐actin reverse: 5′‐GCCGGACTCATCGTACTCC‐3′; IL‐1*β* forward: 5′‐GAAATGCCACCTTTTGACAGTG‐3′, IL‐1*β* reverse: 5′‐TGGATGCTCTCATCAGGACAG‐3′; IL‐6 forward: 5′‐TAGTCCTTCCTACCCCAATTTCC‐3′, IL‐6 reverse: 5′‐TTGGTCCTTAGCCACTCCTTC‐3′; iNOS forward: 5′‐GTTCTCAGCCCAACAATACAAGA‐3′, iNOS reverse: 5′‐GTGGACGGGTCGATGTCAC‐3′; IL‐10 forward: 5′‐CTTACTGACTGGCATGA GGATCA‐3′, IL‐10 reverse: 5′‐GCAGCTCTAGGAGCATGTGG‐3′; TGF‐*β* forward: 5′‐CTCCCGTGGCTTCTAGTGC‐3′, TGF‐*β* reverse: 5′‐GCCTTAGTTTGGACAGGATCTG‐3′; Arg‐1 forward: 5′‐CTCCAAGCCAAAGTCCTTAGAG‐3′, Arg‐1 reverse: 5′‐AGGAGCTGT CATTAGGGACATC‐3′. The polarization state of macrophages in vitro was further confirmed by immunofluorescence assay and flow cytometry. The details of the experimental procedures are the same as above. Mouse‐anti‐iNOS antibody (MA5‐17139, Invitrogen, USA), Alexa Fluor 647‐anti‐CD206 antibody (141 711, BioLegend, USA), Alexa Fluor 568‐anti‐Ms secondary antibody (ab175473, Abcam, Japan) were used for immunofluorescence staining in this section. For flow cytometry, PE‐Cy7‐anti‐CD86 (#25‐0862‐82), APC‐anti‐CD206 (#17‐2062‐82) were all purchased from eBioscience (USA).

### Animals

Male C57 BL/6 mice (21–25 g) aged 6–8 weeks and male ICR mice weighing ≈30 g were all purchased from Shanghai Jiesijie Laboratory Animal, co. Ltd. Mice were maintained in a 12 h/12 h light/dark cycle environment with 22 °C constant temperature and free access to standard laboratory chow and tap water. All animal experiments were approved by the Ethics Committee of Zhongshan Hospital, Fudan University, Shanghai, China and were in compliance with the guidance for the Care and Use of Laboratory Animals published by the National Research Council (U.S.) Institute for Laboratory Animal Research.

### Myocardial Ischemia‐Reperfusion Injury Animal Model

Mice myocardial ischemia‐reperfusion injury model was constructed by left anterior descending coronary artery ligation for 60 min, followed by reperfusion. The injury of mice heart was confirmed by ST‐segment characterized electrocardiogram and left ventricle color alteration.

### Biodistribution and Targeting Specificity of PLM‐miRs In Vivo

To perform pharmacokinetic and biodistribution study, male ICR mice randomly assigned to 3 groups were intravenously administered 200 µL of Cy5 (on miRNA) labeled miRs, MSN‐miRs, LM‐miRs, or PLM‐miRs (1 mg mL^−1^), respectively (*n* = 6). For blood circulating profile, blood samples were collected at different time points (1, 5, 15, and 30 min; 1, 3, 6, 12, 24, and 48 h) via cheek pouch puncture, and the FI of blood samples was measured using a Multiplate Reader (Molecular Devices, USA) with an excitation wavelength of 649 nm and an emission wavelength of 670 nm. In order to reduce individual differences, the experimental data at each time point were calculated relative to the first time point.

To explore the distribution of nanomaterials in major organs and the differences in the accumulation between LM‐miRs and PLM‐miRs in the major organs, including the hearts, liver, spleen, lung, kidney, and brain, after injection at different time points (24 or 72 h post injury), MI/R induced mice were divided into 4 groups randomly (*n* = 6). Two groups were administered with 200 µL DiD (on liposome) labeled LM‐miRs or PLM‐miRs, respectively, at 24 h after injury, and the other two were treated as the same at 72 h post injury. 1 h after the injection of DiD labeled nanoparticles, the FI of the homogenate of the main organs was detected via Multiplate Reader. The FI of the tissue homogenate was adjusted by the following two measures: 1) According to the weight of the obtained tissue, the corresponding volume of PBS was added during the tissue homogenization process. 2) The relative fluorescence intensity (RFI) was determined using the following formula: RFI = FI _heart_/FI _blood_. FI _heart_ is the FI of 200 µL heart tissue homogenate, and FI _blood_ is the FI of 200 µL mouse blood obtained via cheek pouch puncture 1 min after nanoparticles injection.

To further study the biodistribution of the different formulations in major organs after administered at 72 h post injury, MI/R induced C57 BL/6 mice were randomly divided into 3 groups to receive 200 µL PBS, DiD (on liposome) labeled LM‐miRs or PLM‐miRs (1 mg mL^−1^) injection, respectively (*n* = 6). At three time points (1, 3, 24 h) post administration, major organs were harvested and then ex vivo imaged by the IVIS (PerkinElmer, Inc., Waltham, MA). 1 h after the administration, the distribution of nanoparticles in the injured heart was also evaluated by immunofluorescence staining. Briefly, the injured heart was harvest and embedded in optimal cutting temperature compound (Sakura Finetek, Japan), and then frozen in liquid nitrogen before being cut into 8‐um cryosections. Tissue sections were either immediately processed for subsequent staining, or frozen at −20 °C for further use. For immunofluorescence staining, the obtained tissue sections were first fixed in acetone for 10 min, and then washed three times with PBS, each time for 5 min. The washed tissue sections were blocked with 3% BSA for 1 h, and then incubated with a specific primary antibody, Rabbit‐anti‐cardiac troponin T (15513‐1‐AP, ProteinTech, USA), overnight at 4 °C. After washing three times with PBS, each time for 5 min, it was stained with fluorescently labeled secondary antibody for 1 h, followed by DAPI to indicate the nucleus. Alexa Fluor 488‐anti‐Rabbit IgG (ab150077) secondary antibody was purchased from Abcam (Japan).

Flow cytometry and immunofluorescence staining were conducted to further explore the underlying mechanism of the selective accumulation of PLM‐miRs in the injured heart. MI/R induced C57 BL/6 mice were randomly divided into 2 groups (*n* = 6), which were injected with 200 µL DiD (on liposome) labeled LM‐miRs or PLM‐miRs, respectively, at 72 h post injury. 30 min after the injection, the blood of the mice was stained with specific antibodies, FITC‐anti‐CD45 (#561 867, eBioscience, USA), PerCP‐Cy5.5‐anti‐CD11b (#45‐0112‐82, eBioscience, USA) and PE‐Cy7‐anti‐Ly6C (#560 593, BD Pharmingen, USA), and then treated with RBC Lysis Buffer (Invitrogen, USA) for 5 min. After centrifugation at 1000 rpm for 5 min, the cells were resuspended in Staining Buffer, and fluorescence signals were detected by BD FACS Aria III. To evaluate the aggregation of nanoparticles in local monocytes/macrophages in the heart, we divided MI/R induced mice into 4 groups randomly (*n* = 6). Two groups were administered with 200 µL DiD (on liposome) labeled LM‐miRs or PLM‐miRs, respectively, at 24 h after injury, and the other two were given the same treatment at 72 h post injury. None‐cardiomyocytes single‐cell suspension of tissues in the marginal area of the injury was obtained by using Multi Tissue Dissociation Kit 2 (Miltenyi Biotec, USA) according to the instructions. Briefly, after cut into small pieces (1–2 mm^3^), harvested heart tissue was digested in Enzyme Mix at 37 °C for 15 min, followed by mechanical agitation through the Program Multi_G of the gentle MACS Dissociator (Miltenyi Biotec, USA), and then repeated once. After filtered through MACS SmartStrainer (70 um), the resulting sample was centrifuged at 600 g for 5 min to separate singe cells. The cell pellet of one sample was resuspended in 200 uL staining buffer. Follow‐up staining and detection process are the same as described previously. Specific primary antibodies, FITC‐anti‐CD45 (#561 867, eBioscience, USA), PerCP‐Cy5.5‐anti‐CD11b (#45‐0112‐82, eBioscience, USA), were used.

To further explore the pathway of PLM‐miRs to reach the MI area, we performed a colocalization analysis on the nanomaterials and monocytes/macrophages in the injured area by using immunofluorescence staining. The staining process is the same as described above, and Rat‐anti‐F4/80 antibodies (ab6640, Abcam, Japan), and Alexa Fluor 488‐anti‐Rat secondary antibody (ab150165, Abcam, Japan) were used. Fluorescence signals were detected by CLSM and colocalization analysis of the images was performed by ImageJ.

### Polarization State Analysis of Macrophages In Vivo

In vivo, the randomly divided three groups (*n* = 6) of MI/R induced mice were treated with 200 µL PBS, LM‐miRs or PLM‐miRs, respectively. Two days after administration (the 5th d post injury), phenotypes of heart macrophages were detected by immunofluorescence assay and flow cytometry as well. All experimental procedures are the same as previously described. Alexa Fluor 647‐anti‐CD206 antibody (141 711, BioLegend, USA), Rat‐anti‐F4/80 antibody (ab6640, Abcam, Japan) and Alexa Fluor 488‐anti‐Rat secondary antibody (ab150165, Abcam, Japan) were used for immunofluorescence staining in this section. For flow cytometry, FITC‐anti‐CD45 (#561 867), PerCP‐Cy5.5‐anti‐CD11b (#45‐0112‐82), PE‐anti‐F4/80 (#565 410), PE‐Cy7‐anti‐CD86 (#25‐0862‐82), APC‐anti‐CD206 (#17‐2062‐82) were all purchased from eBioscience (USA). ELISA were adopted to detect the concentration of cytokines (TNF‐*α*, IL‐1*β*, TGF‐*β*, and IL‐10) in cardiac tissue homogenate after PBS, LM‐miRs or PLM‐miRs treatment according to the manufacturer's instruction. Before the detection, the BCA assay was used to quantify the protein concentration of the tissue homogenate, and the subsequent detection results were also corrected according to the corresponding protein concentration.

### Cardiac Protection Efficiency of PLM‐miRs

MI/R induced C57 BL/6 mice were blindly divided into three groups (*n* = 6) and the groups were injected 200 µL PBS, LM‐miRs or PLM‐miRs, respectively, through the tail vein on the third day after the procedure. Cardiac geometry structure and function were assessed using the 2D guided M‐mode echocardiography (Visual Sonics Vevo 770, Canada) 1 day before the procedure and 4 weeks after the treatments. After depilation, mice were anesthetized with controlled isoflurane flow to maintain the heart rate around 450 beats min^−1^. The parasternal long‐axis view was obtained and LVEF, fraction shortening, LVESV, and LVEDV were calculated and analyzed. The echocardiographer was blind to the treatment protocol of mice and each measurement index was averaged by six consecutive cardiac cycles. After that, the mouse hearts were harvested to make 5‐um‐thick paraffin sections in several different layers. Masson staining of paraffin sections was used to determine the infarct size and the degree of remodeling fibrosis. To explore whether the regulation of the polarization state of macrophages by PLM‐miRs contributes to the maturation of sprouting vessels, immunofluorescence staining against CD31, a typical mature endothelial marker, were conducted at 7 d post‐MI/R (the 5th day after PLM‐miRs treatment). Rabbit‐anti‐CD31 (ab28364, Abcam, Japan) were used and the staining process is the same as described above.

### Cytotoxicity Assays of PLM‐miRs

The cytotoxicity of PLM‐miRs to BMDM and H9C2 was assessed using a CCK‐8 kit (Beyotime, China) according to the manufacturer's protocol, and the absorbances were measured at 450 nm by an EPOCH 2 microplate reader (BioTek, USA).

### Biosafety Analysis of PLM‐miRs

Considering the safety of immunity, C57 B/L mice were randomized and treated with 200 µL PLM‐miRs or PBS (*n* = 6). The serum level of inflammatory cytokines, TNF‐*α* and IL‐1*β*, and general antibodies, IgG and IgM, were all quantified by using ELISA kits (Biolegend, USA) according to the manufacturer's protocols. For organ toxicity, biochemical tests of liver (aspartate aminotransferase, AST; alanine aminotransferase, ALT) and renal function (creatinine, CREA; urea nitrogen, UREA) were performed on the serum of mice after treated with PBS or PLM‐miRs. Histopathological changes in major organs were also evaluated by HE staining. To assess the potential impact of PLM‐miRs on coagulation function, whole blood anticoagulated with sodium citrate was analyzed by APTT, PT, and Fbg.

### Statistical Analysis

All quantitative data are presented as means ± standard deviation (SD) of at least triplicate measurements. Two‐tailed Student's *t*‐test was used for comparison between two groups. One‐way analysis of variance (ANOVA) followed by Bonferroni test was used for statistics between multiple groups. The difference between groups was considered none statistically significant for NS, statistically significant for *P < 0.05, very significant for **P < 0.01, and the most significant for ***P < 0.001. Statistical and graph analysis were performed using SPSS Statistics 26.0 (IBM, USA) and GraphPad Prism 7.0 (GraphPad Software, USA), respectively.

## Conflict of Interest

The authors declare no conflict of interest.

## Supporting information

Supporting Information

## Data Availability

Research data are not shared.
