# Supplementary material for: Platelet‐Like Fusogenic Liposome‐Mediated Targeting Delivery of miR‐21 Improves Myocardial Remodeling by Reprogramming Macrophages Post Myocardial Ischemia‐Reperfusion Injury
Source: Adv Sci (Weinh). 2021 Jun 17;8(15):2100787. doi: 10.1002/advs.202100787 (PMC8336489; doi:10.1002/advs.202100787)
Supplement: Supplementary file 1 — Supporting Information [file ADVS-8-2100787-s001.pdf]

© 2021 Wiley-VCH GmbH

ADVANCED SCIENCE Open Access

Supporting Information

for *Adv. Sci.,* DOI: 10.1002/advs.202100787

Mimicking Platelet-monocyte Aggregation as a Targeting Strategy to Reprogram Macrophages Post Myocardial Ischemia-reperfusion Injury

Haipeng Tan, Yanan Song, Jing Chen, Ning Zhang, Qiaozi Wang, Qiyu Li, Jinfeng Gao, Hongbo Yang, Zheng Dong, Xueyi Weng, Zhengmin Wang, Dili Sun, Wusiman Yakufu, Zhiqing Pang, Zheyong Huang*, Junbo Ge**

Mimicking platelet-monocyte aggregation as a targeting strategy to reprogram macrophages post myocardial ischemia-reperfusion injury

Haipeng Tan, Yanan Song, Jing Chen, Ning Zhang, Qiaozi Wang, Qiyu Li, Jinfeng Gao, Hongbo Yang, Zheng Dong, Xueyi Weng, Zhengmin Wang, Dili Sun, Wusiman Yakufu, Zhiqing Pang, Zheyong Huang*, Junbo Ge**

Department of Cardiology, Zhongshan Hospital, Fudan University, Shanghai Institute of Cardiovascular Diseases.

School of Pharmacy, Fudan University, Key Laboratory of Smart Drug Delivery, Ministry of Education.

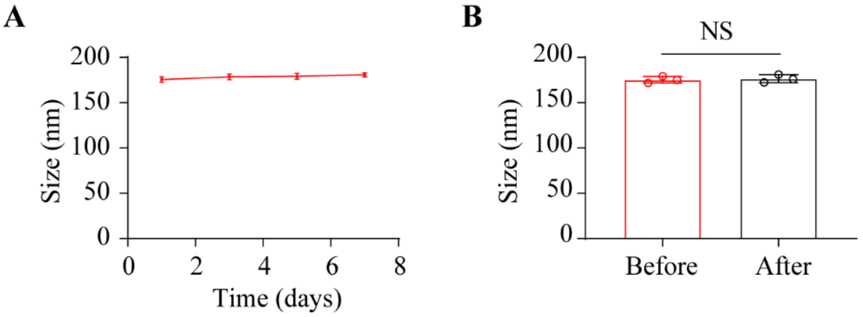

Supplementary Figure 1. *In vitro* stability of PLM-miRs. (A) Size of PLM-miRs Over time in PBS detected by DLS (n=3). (B) Size of PLM-miRs before or after repeated freezing and thawing cycles detected by DLS. Results are presented as mean ± SD. $^{NS}P > 0.05$.

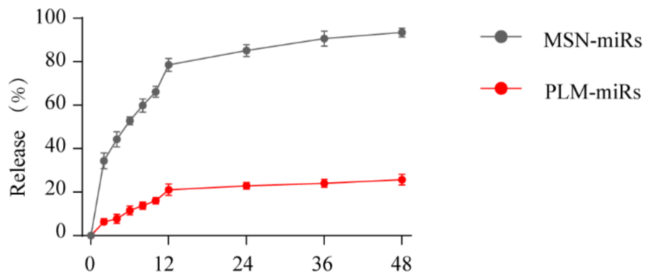

Supplementary Figure 2. miRNA release from MSN-miRs or PLM-miRs at 37℃ plus pH 7.4 over time (n = 3). Results are presented as mean ± SD.

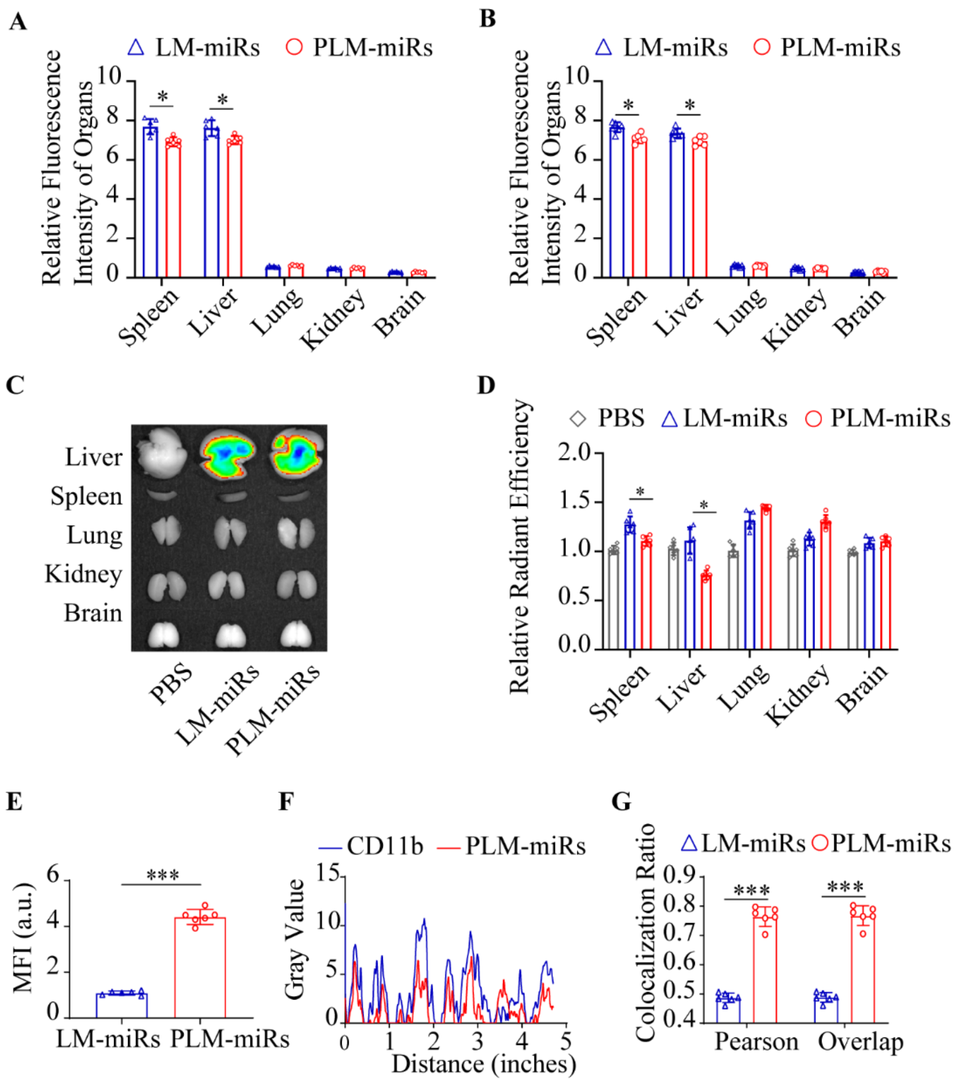

Supplementary Figure 3. Distribution of LM-miRs and PLM-miRs in major organs after intravenous injection. At two time points post injury, (**A**) 24 h and (**B**) 48 h, the fluorescence intensity of the main organs tissue homogenate of MI/R induced mice was detected after the injection of DiD-labeled LM-miRs or PLM-miRs (n = 6). (**C**) IVIS images of major organs of MI/R induced mice after treated with PBS, DiD (on liposome) labeled LM-miRs or PLM-miRs. (**D**) Quantitative analysis of the accumulation of LM-miRs and PLM-miRs in major organs of MI/R induced mice based on images in (C) (n = 6). (**E**) Quantification of the accumulation of LM-miRs and PLM-miRs in the MI/R injured heart based on the CLSM images in Fig.5E (n=6). (**F**) The plot profile, (**G**) pearson's correlation and overlap coefficient were quantified after colocalization analysis of images from Fig.5F via Image J (n=6). Results are presented as mean ± SD. $^{NS}P > 0.05$, $*P < 0.05$, $**P < 0.01$, $***P < 0.001$.

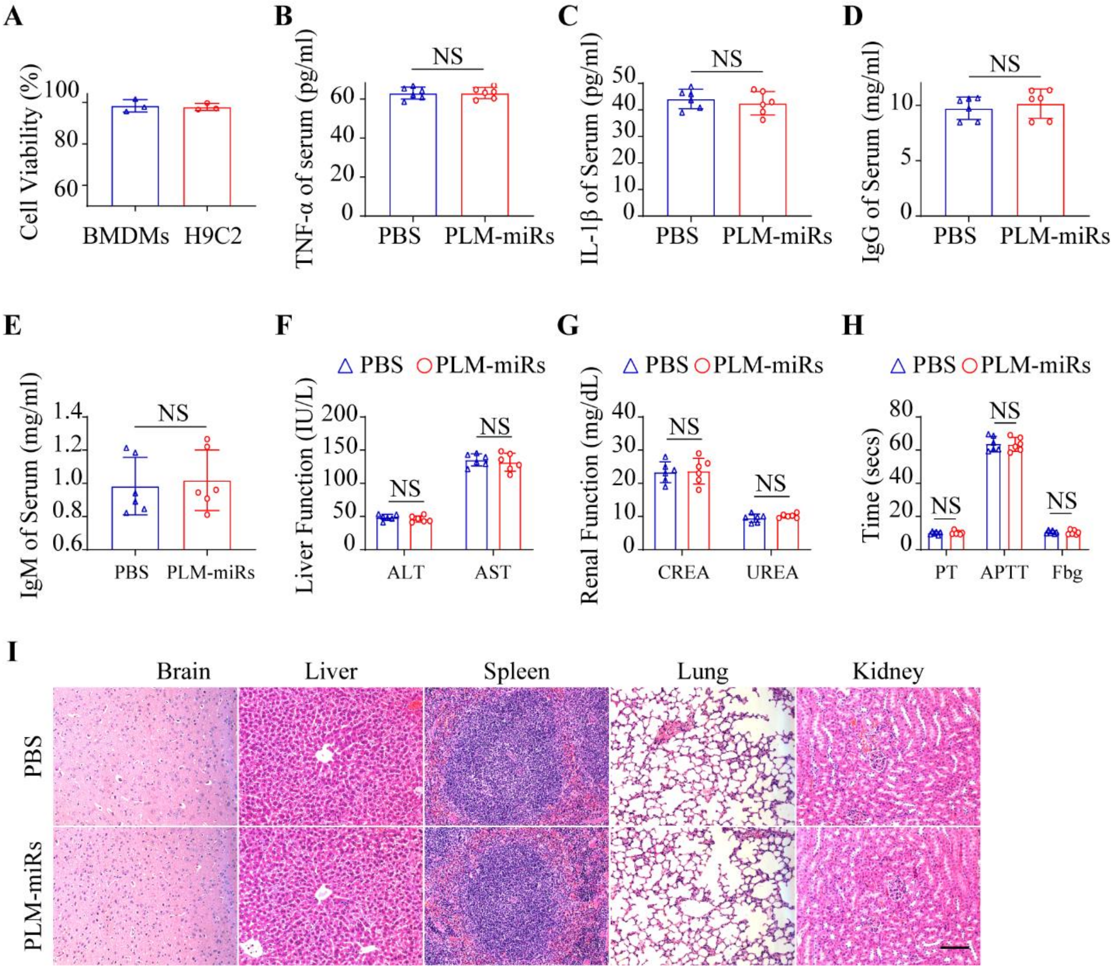

Supplementary Figure 4. Biosafety verification of PLM-miRs. (**A**) Relative cell viability of BMDMs and H9C2s as evaluated by the CCK8 assay after treated with PLM-miRs (n = 3). (**B** and **C**) Serum concentration of inflammatory cytokines (IL-1β, TNF-α) of healthy mice detected by ELISA assay at 3 d post administration of PBS or PLM-miRs (n=6). (**D** and **E**) ELISA assay of immune response indicated by serum level of general IgG and IgM of healthy mice with or without PLM-miRs treatment (n = 6). Biochemical test of (**F**) liver and (**G**) renal function of healthy mice after PBS or PLM-miRs administered (n = 6). (**I**) Histology characteristics of major organs of PBS or PLM-miRs treated healthy mice were detected by HE staining. Scalar bar, 100 μm. Results are presented as mean ± SD. $^{NS}P > 0.05$.

Tandem Mass Tag^{TM} (TMT) quantitative proteomics analysis

Materials and Methods

1. Total Protein Extraction

Sample was lysed with lysis buffer which containing 100 mM NH_4HCO_3(pH 8), 8 M Urea and 0.2% SDS, followed by 5 min of ultrasonication on ice. The lysate was centrifuged at 12000 g for 15 min at 4℃ and the supernatant was transferred to a clean tube. Extracts from each sample were reduced with 10 mM DTT for 1 h at 56℃, and subsequently alkylated with sufficient iodoacetamide for 1 h at room temperature in the dark. Then samples were completely mixed with 4 times volume of precooled acetone by vortexing and incubated at -20℃ for at least 2 h. Samples were then centrifuged and the precipitation was collected. After washing twice with cold acetone, the pellet was dissolved by dissolution buffer, which containing 0.1 M triethylammonium bicarbonate (TEAB, pH 8.5) and 6 M Urea.

2. Protein Quality Test

BSA standard protein solution was prepared according to the instructions of Bradford protein quantitative kit, with gradient concentration ranged from 0 to 0.5 g/L. BSA standard protein solutions and sample solutions with different dilution multiples were added into 96-well plate to fill up the volume to 20 ul, respectively. Each gradient was repeated three times. The plate was added 180 ul G250 dye solution quickly and placed at room temperature for 5 minutes, the absorbance at 595 nm was detected. The standard curve was drawn with the absorbance of standard protein solution and the protein concentration of the sample was calculated. 20 ug of the protein sample was loaded to 12% SDS-PAGE gel electrophoresis, wherein the concentrated gel was performed at 80 V for 20 min, and the separation gel was performed at 120 V for 90 min. The gel was stained by coomassie brilliant blue R-250 and decolored until the bands were visualized clearly.

3. TMT Labeling of Peptides

120 ug of each protein sample was taken and the volume was made up to 100 ul with dissolution buffer, 1.5 ug trypsin and 500 ul of 100 mM TEAB buffer were added, sample was mixed and digested at 37 °C for 4h. And then, 1.5 ug trypsin and $CaCl_2$ were added, sample was digested overnight. Formic acid was mixed with digested sample, adjusted pH under 3, and centrifuged at 12000 g for 5 min at room

temperature. The supernatant was slowly loaded to the C18 desalting column, washed with washing buffer (0.1% formic acid, 3% acetonitrile) 3 times, then eluted by some elution buffer (0.1% formic acid, 70% acetonitrile). The eluents of each sample were collected and lyophilized. 100 ul of 0.1 M TEAB buffer was added to reconstitute, and 41 ul of acetonitrile-dissolved TMT labeling reagent was added, sample was mixed with shaking for 2 h at room temperature. Then, the reaction was stopped by adding 8% ammonia. All labeling samples were mixed with equal volume, desalted and lyophilized.

4. Separation of fractions

Mobile phase A (2% acetonitrile, adjusted pH to 10.0 using ammonium hydroxide) and B (98% acetonitrile) were used to develop a gradient elution. The lyophilized powder was dissolved in solution A and centrifuged at 12,000 g for 10 min at room temperature. The sample was fractionated using a C18 column (Waters BEH C18 4.6×250 mm, 5 um) on a Rigol L3000 HPLC system, the column oven was set as 50°C. The detail of elution gradient was shown in Table 1. The eluates were monitored at UV 214 nm, collected for a tube per minute and combined into 10 fractions finally. All fractions were dried under vacuum, and then, reconstituted in 0.1% (v/v) formic acid (FA) in water.

5. LC-MS/MS Analysis

For transition library construction, shotgun proteomics analyses were performed using an EASY-nLCTM 1200 UHPLC system (Thermo Fisher) coupled with an Q Exactive HF-X mass spectrometer (Thermo Fisher) operating in the data-dependent acquisition (DDA) mode. 1 ug sample was injected into a home-made C18 Nano-Trap column (2 cm×75 um, 3 um). Peptides were separated in a home-made analytical column (15 cm×150 um, 1.9 um), using a linear gradient elution. The separated peptides were analyzed by Q Exactive HF-X mass spectrometer (Thermo Fisher), with ion source of Nanospray Flex™（ESI）, spray voltage of 2.3 kV and ion transport capillary temperature of 320°C. Full scan ranging from m/z 350 to 1500 with resolution of 60000 (at m/z 200), an automatic gain control (AGC) target value was 3×106 and a

maximum ion injection time was 20 ms. The top 40 precursors of the highest abundant in the full scan were selected and fragmented by higher energy collisional dissociation (HCD) and analyzed in MS/MS, where resolution was 30000 (at m/z 200) for 6 plex, the automatic gain control (AGC) target value was 5×104 the maximum ion injection time was 54 ms, a normalized collision energy was set as 32%, an intensity threshold was 1.2×105, and the dynamic exclusion parameter was 20 s.

6. Data analysis

6.1. The identification and quantitation of protein

The resulting spectra from each run were searched separately against homo_sapiens_uniprot_2020_7_2. fasta (192320 sequences) database by the search engines: Proteome Discoverer 2.2 (PD 2.2, Thermo). The searched parameters are set as follows: mass tolerance for precursor ion was 10 ppm and mass tolerance for product ion was 0.02 Da. Carbamidomethyl was specified as fixed modifications, Oxidation of methionine (M) and TMT plex were specified as dynamic modification, acetylation and TMT plex were specified as N-Terminal modification in PD 2.2. A maximum of 2 miscleavage sites were allowed.

In order to improve the quality of analysis results, the software PD 2.2 further filtered the retrieval results: Peptide Spectrum Matches (PSMs) with a credibility of more than 99% was identified PSMs. The identified protein contains at least 1 unique peptide. The identified PSMs and protein were retained and performed with FDR no more than 1.0%. The protein quantitation results were statistically analyzed by T-test. The proteins whose quantitation significantly different between experimental and control groups, $p < 0.05$ and $|\log2FC| > 0.26$ (ratio > 1.2 or ratio < 0.83 [fold change, FC]), were defined as differentially expressed proteins (DEP).

6.2. The functional analysis of protein and DEP

Gene Ontology (GO) and InterPro (IPR) functional analysis were conducted using the interproscan program against the non-redundant protein database (including Pfam, PRINTS, ProDom, SMART, ProSite, PANTHER),[1] and the databases of COG (Clusters of Orthologous Groups) and KEGG (Kyoto Encyclopedia of Genes and Genomes) were used to analyze the protein family and pathway. DPEs were used for

Volcanic map analysis, cluster heat map analysis and enrichment analysis of GO, IPR and KEGG.[2] The probable protein-protein interactions were predicted using the STRING-db server (http://string.embl.de/).[3]

7. Supplementary Reference:

[1] P. Jones, D. Binns, H. Y. Chang, M. Fraser, W. Li, C. McAnulla, H. McWilliam, J. Maslen, A. Mitchell, G. Nuka, S. Pesseat, A. F. Quinn, A. Sangrador-Vegas, M. Scheremetjew, S. Y. Yong, R. Lopez, S. Hunter, *Bioinformatics* **2014**, *30*, 1236.

[2] W. Huang-da, B.T. Sherman, and R.A. Lempicki, *Nucleic Acids Res* **2009**, *37*, 1.

[3] A. Franceschini, D. Szklarczyk, S. Frankild, M. Kuhn, M. Simonovic, A. Roth, J. Lin, P. Minguez, P. Bork, C. V. Mering, L. J. Jensen, *Nucleic Acids Res* **2013**, *41*, D808.
